# Comparing transabdominal and transvaginal cervical length measurements at mid‐trimester fetal anomaly scan: The impact of bladder fullness and lower uterine contractions

**DOI:** 10.1002/ajum.12409

**Published:** 2024-08-29

**Authors:** Heidi Beaver, Valeria Lanzarone, Gary KK Low

**Affiliations:** ^1^ Christopher Kohlenberg Department of Perinatal Ultrasound Nepean Hospital, Nepean Blue Mountain Local Health District Kingswood New South Wales Australia; ^2^ Research Operations Nepean Hospital, Nepean Blue Mountain Local Health District Kingswood New South Wales Australia

**Keywords:** cervical length screening, mid‐trimester fetal anomaly scan, transabdominal, transvaginal, ultrasound, uterine contraction

## Abstract

**Purpose:**

To assess the effects of bladder fullness and lower uterine contractions ultrasound on transabdominal and transvaginal cervical length measurements at the mid‐trimester fetal anomaly scan (FAS).

**Methods:**

Transabdominal and transvaginal cervical length measurements from 925 mid‐trimester FAS examinations were retrospectively analysed. Images were assessed for lower uterine contraction and bladder fullness using a novel qualitative assessment. Bland–Altman plots and single‐score interclass correlation (ICC) were used to determine correlation between transabdominal and transvaginal measurements. Sensitivity and specificity of transabdominal cut‐offs were calculated.

**Results:**

Transabdominal and transvaginal measurements of the cervix correlated poorly (ICC 0.306). An overfilled bladder and lower uterine contractions on average increased the length of transabdominal cervical length measurements. Removing these variables did not significantly improve correlation between transabdominal and transvaginal measurements of the cervix but resulted in an improved sensitivity of transabdominal assessment to detect a clinically relevant short cervix.

**Discussion:**

Resolving the confounding factors of an overfilled bladder and lower uterine contractions can help improve the our ability to detect a short cervix on transabdominal ultrasound. Our data set supported a two‐stage approach to cervical length screening which would allow 100% sensitivity when a cut‐off of ≤35 mm is used on transabdominal ultrasound and would limit the need for transvaginal scanning to approximately 39% of patients. This cut‐off is in line with the findings of other studies. The low prevalence of short cervix in our study did however make it difficult to extrapolate reliable calculations.

**Conclusion:**

Although transabdominal measurements correlate poorly with transvaginal measurements of the cervix, we demonstrated an improved sensitivity for detecting a short cervix using a transabdominal approach when no contractions or overfilled bladder is present. This potential could be explored in a future study with a larger sample size.

## Introduction

Preterm birth contributes to morbidity and mortality of babies especially when prematurity occurs prior to 32 weeks gestation.^1^, ^2^, ^3^ In Australia, 8.7% of births are preterm (<37 week gestation) and 19% of preterm births are <32 weeks gestation.^4^ Preterm babies make up 85% of stillbirths and perinatal death among babies born at 20–27 weeks is 694 per 1000 births.^4^ Morbidity related to preterm birth affects quality of life and leads to economic strain on the Australian health care system.^5^ Babies born preterm spend on average more than twice as long in hospital than those delivered at term and account for 80% of babies requiring Special Care Nursery or Neonatal Intensive Care Unit care.^4^ Preterm birth also leads to emotional stress on parents. Transvaginal cervical length screening has been shown to help identify pregnancies at risk of preterm birth, allowing interventions to be offered.^6^, ^7^, ^8^

Most studies on cervical length screening have been performed in the mid‐trimester, and this is an effective time for assessing cervical length and risk of preterm labour. Transvaginal cervical measurement (TVCM) is considered the gold standard for measuring cervical length due to its higher resolution, fewer artefacts and lower likelihood of interference from overlying organs.^9^ A TVCM cut‐off of 25 mm is commonly used to define a short cervix.^10^ At 19 weeks gestation, it represents the lowest one percentile of charted length^11^ and predicts a likelihood ratio of 4.3 for preterm birth <35 weeks^12^. There are many studies of short cervix and preterm birth using TVCM but few studies have looked at transabdominal cervical measurements (TACM) and preterm birth. It is not clear whether TACM is accurate enough to effectively screen for preterm birth but it has obvious advantages in terms of scanning time^13^ and reduction in patient uneasiness. Cho *et al*.^14^ found good correlation between TACM and premature birth when compared to TVCM studies; however, they found the cervix could not be measured transabdominally 20% of the time and this rate was higher when a short cervix was present.

Previous studies looking at the difference between TACM and TVCM have demonstrated discrepancies in measurements obtained between the techniques, some stating TACM are shorter than TVCM^15^, ^16^ and others longer.^17^, ^18^ Cho *et al*.^14^ found no significant difference between TACM and TVCM when performed immediately after each other and with the same degree of maternal bladder fullness.

As Cho *et al*.^14^ state, ‘The aim of cervical length assessment is not to establish real cervical lengths but to predict preterm birth, it is important to be able to accurately identify a substantially short cervical length using this technique (transabdominal sonography)’, the issue then is not correlation but how to best identify a short cervix. It has often been stated that TACM is susceptible to overmeasuring the cervix by elongation due to a full bladder or probe pressure.^19^ Poor transabdominal image quality issues due to body habitus, poor landmark identification, lower segment contractions and fetal interference have been raised as potential problems.^20^ Rhoades *et al*.^21^ report that adequate transabdominal images could only be obtained in one of five cases.

Bladder filling appears to be a crucial variable. Several studies have demonstrated that a full bladder overestimates cervical length, but the definition of a full bladder has varied between studies.^18^, ^22^, ^23^ Hernandesz *et al*.^22^ does not define how full the bladder was at the time of scanning but simply state, ‘if the bladder was not sufficiently full to provide an acoustic window, the examination was delayed until visualization of the cervix was achieved’. Friedman *et al*.^24^ instructed patients to refrain from voiding for 2 h prior to the exam but how full the bladder was at time of scanning is not defined. Marren *et al*.^18^ classified the bladder as full or empty with the degree of fullness measured as a volume. Marren *et al*.^18^ demonstrated an increase in overestimation of the cervix with increasing bladder fullness measured by volume, stating ‘For every 100 mL increase in bladder volume, the cervical length was artificially lengthened 2.4 mm’. Specific bladder filling can be time consuming and is not practicable. This article outlines a novel method of assessing bladder fullness that is quick and easy and can help limit inaccuracies in TACM.

Uterine or myometrial contractions have been noted on obstetric ultrasound as early as the 1970s.^25^ A uterine contraction on ultrasound can best be defined as a transient focal myometrial thickening. This thickening, sometimes described as a bulge or mass, is typically isoechoic and homogenous and will resolve or change in configuration within 30–60 min. Typically, they bulge inwards and are limited to a region of the myometrium. The effect of lower uterine contractions on cervical length is yet to be explored in literature. This study compares TACM with TVCM obtained at the same mid‐trimester fetal anomaly scan (FAS) in a large group of unselected patients to assess correlation and the effects of bladder fullness and lower uterine contractions on measurements. Measurements collected in the absence of these confounders were sub‐analysed to determine if there was better correlation and/or accuracy in detecting a short cervix transabdominally.

## Methods

This study was a retrospective audit looking at cervical lengths obtained at routine mid‐trimester FAS during the time period between January 2019 and June 2020 at the Christopher Kohlenberg Department of Perinatal Ultrasound, Nepean Hospital.

The cohort was a mixture of low‐risk and high‐risk tertiary referred patients. The presence of fetal anomaly or pre‐existing risk factors were not exclusions. With very few exceptions these were outpatient examinations. Scans were performed by eight accredited medical sonographers (AMSs) or obstetricians using a RIC 5–9‐D transvaginal probe and a C4‐8‐D or RAB 6‐D transabdominal probe on GE E10, 8 and 6 machines (GE Healthcare, Milwaukee, WI, USA). During most of this time, it was standard practice to image the cervix and lower segment transabdominally as part of the routine mid‐trimester FAS examination and record a TACM. The examinations also included a brief transvaginal scan for the purpose of recording TVCM, hence both TACM and TVCM were generally available for retrospective analysis. From March 2020, transvaginal ultrasound was not offered routinely at the FAS in order to reduce contact time with patients due to the COVID‐19 pandemic and this meant that a small cohort had only TACM recorded.

Data collected included: gestational age, TACM, classification of bladder fullness, TVCM and presence of visible lower uterine contractions. Where multiple measurements were taken transabdominally and/or transvaginally, an independent AMS assessed images determining the image which most adequately displayed landmarks (internal and external ora) and the shortest measurements were used for the statistical analysis. A single straight‐line measurement was used in accordance with the International Society of Ultrasound in Obstetrics and Gynecology guidelines.^26^ Curved 2DTrace measurements were excluded. Patients, where neither TACM nor TVCM was acquired, were excluded from analysis. In several patients, imaging revealed the cervix to be subjectively very short or open on transabdominal views but a TACM was not recorded. These patients were classified as having a TACM <10 mm for analytical purposes. Measurements were categorised according to bladder fullness and presence of uterine contractions.

**Figure 1:**
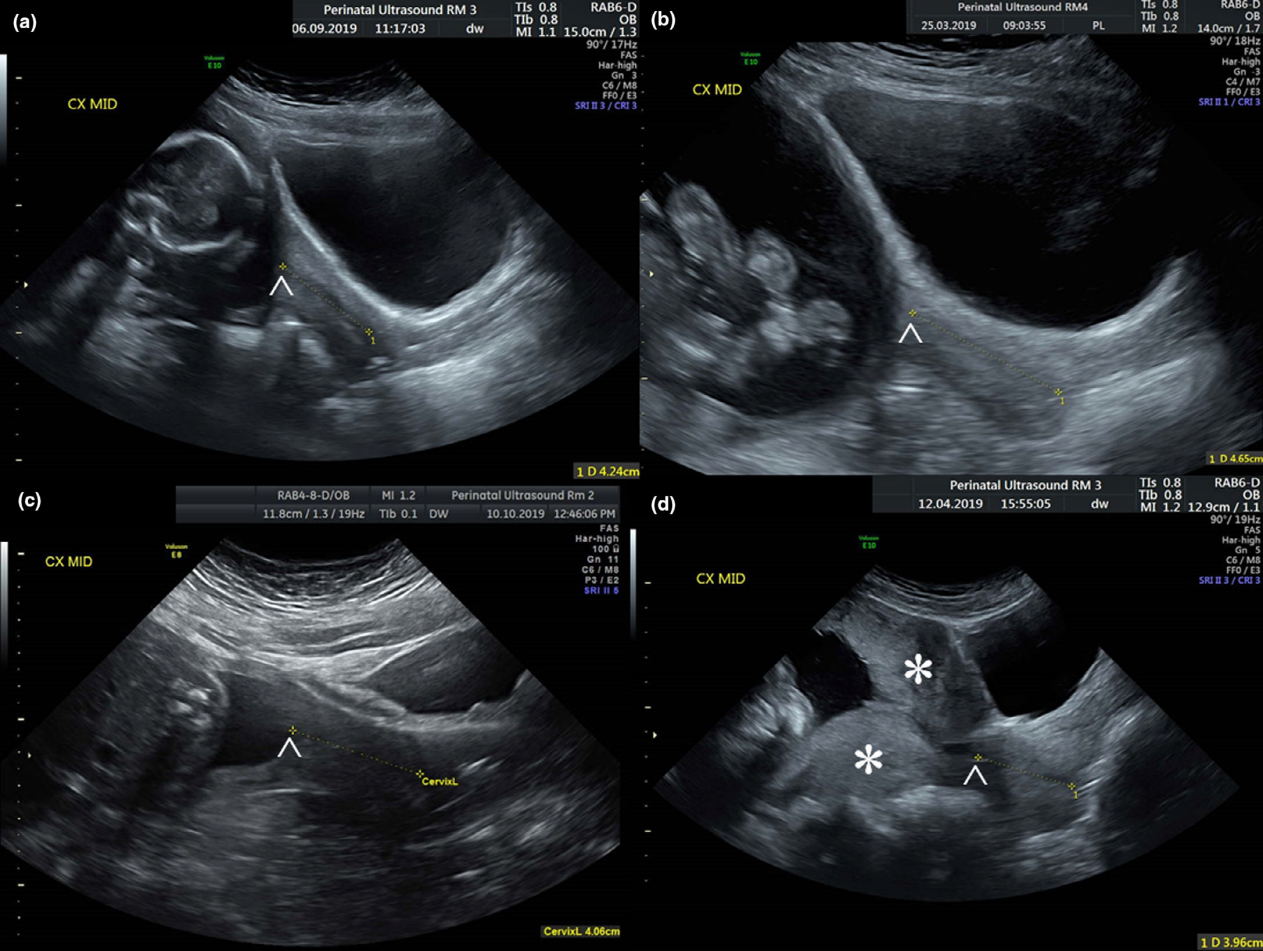
Bladder fullness: (a) bladder classified as overfilled with cephalad margin at the level of the internal os, (b) bladder classified as overfilled with cephalad margin above the level of the internal os, (c) bladder classified as not overfilled with cephalad margin below the level of the internal os, (d) bladder classified as overfilled with cephalad margin at the level of the internal os and lower uterine contraction present. *Lower uterine contraction, ^Internal os.

**Figure 2:**
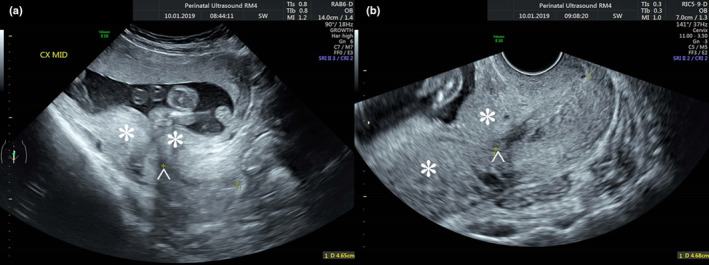
Lower uterine contractions seen: (a) transabdominally and (b) transvaginally. *Lower uterine contraction, ^Internal os.

It was routine practice to advise patients to arrive for their scan with a comfortable bladder. This means the degree of bladder filling observed during TACM was highly variable. TVCM was routinely acquired after instructing the patients to empty their bladders. An easy visual qualitative measurement was devised by the principal investigator to retrospectively determine bladder ‘overfilling’ on the images obtained. This novel assessment classified a bladder as overfilled when the cephalad margin of the bladder wall was at the level of or more cephalic to the internal os of the cervix (Figure 1). The internal os is generally identified by the characteristic sharp junction between cervical canal and lower uterine cavity, the echogenic endocervical mucosa or with reference to the position of the uterine arteries. TACM were sub‐categorised according to the presence or absence of an overfull bladder according to this method. TVCM were all sub‐categorised as measured in the absence of an overfilled bladder. Sub‐categories were also created for TACM and TVCM according to the presence or absence of lower uterine contractions as assessed subjectively by the principal investigator on the images obtained (Figure 2).

Welch's two‐sample *t*‐test and box plots in R 4.2 were used to analyse different categories to determine the effects of bladder fullness and contractions. Bland–Altman plots, interclass correlation coefficients (ICC) estimates and their 95% confident intervals (CI) were calculated using R 4.1.1 based on an absolute‐agreement, two‐way mixed‐effects model to compare and test correlation of TACM and TVCM in corresponding groups of different categories.

The group where TACM were obtained without evidence of an overfilled bladder and/or lower uterine contraction were further compared with corresponding TVCM. The accuracy of TACM overall, and in the group without confounders, was calculated using different TACM cut‐offs to determine if a certain cut‐off produced an adequate sensitivity and specificity for a screening test for a short cervix. A receiver operator curve (ROC) was produced in Microsoft Excel.

### Ethics approval

Ethics approval was obtained via Research Ethics and Governance Information System. The project was determined to meet the requirements of the National Statement on Ethical Conduct in Human Research (2007) and approval was given by the Nepean Blue Mountains Local Health District Human Research Ethics Committee. In this retrospective study, deidentified data was used and the study was found exempt to obtaining patient consent.

## Results

**Figure 3:**
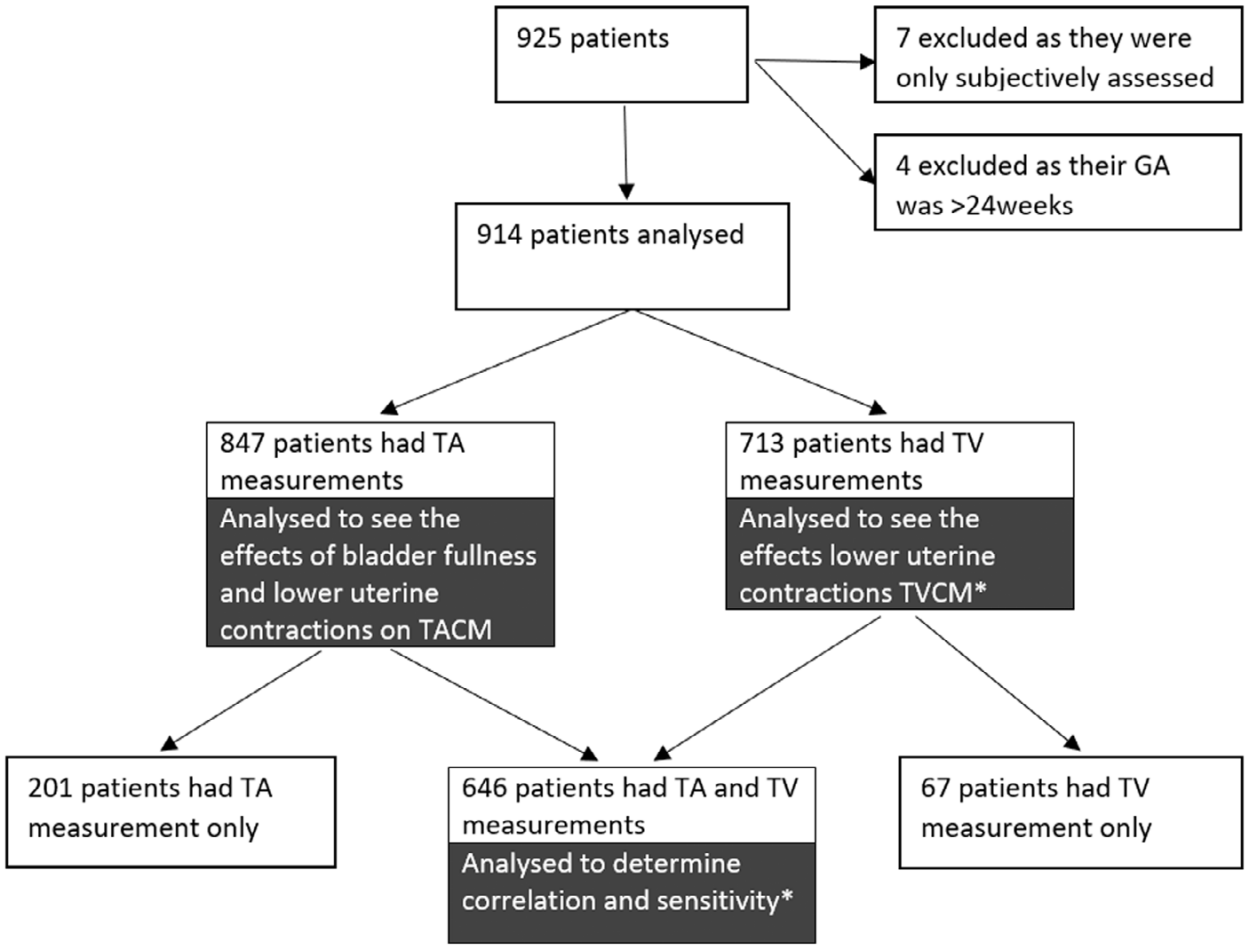
TA and TV measurements analysed. * Two excluded from analysis as TVCM was measured with curved trace instead of straight line. TA, transabdominal; TACM, transabdominal cervical measurements; TV, transvaginal; TVCM, transvaginal cervical measurement.

Investigation was conducted on 925 patients from the Viewpoint archive of the department. Of these 925 patients, 7 were excluded as neither TACM nor TVCM were recorded and 4 were excluded as their gestation was >24 weeks. There were 914 patients included in the analysis. Of these, a total of 646 patients had both a TACM and TVCM, 201 had TACM only (TVCM not offered or declined) and 67 had TVCM only (Figure 3). Of 811 women scanned prior to a change in protocol due to the COVID‐19 pandemic, 147 declined transvaginal scanning (18%). Two patients were excluded from TVCM analysis as they were measured with a curved trace instead of a linear measurement. This provided 644 patients for comparison between TVCM and TACM and 711 patients for sub‐analysis of effects of contraction on TVCM. There were 847 patients who had TACM analysed to determine the effects of a full bladder and lower uterine contractions.

The age range of the patients analysed was between 17 and 43 years old (mean 29.57, median 29). The average age of mothers giving birth in Australia in 2017 was comparable (mean 30.6, median 31).^4^ Parity was not recorded.

**Figure 4:**
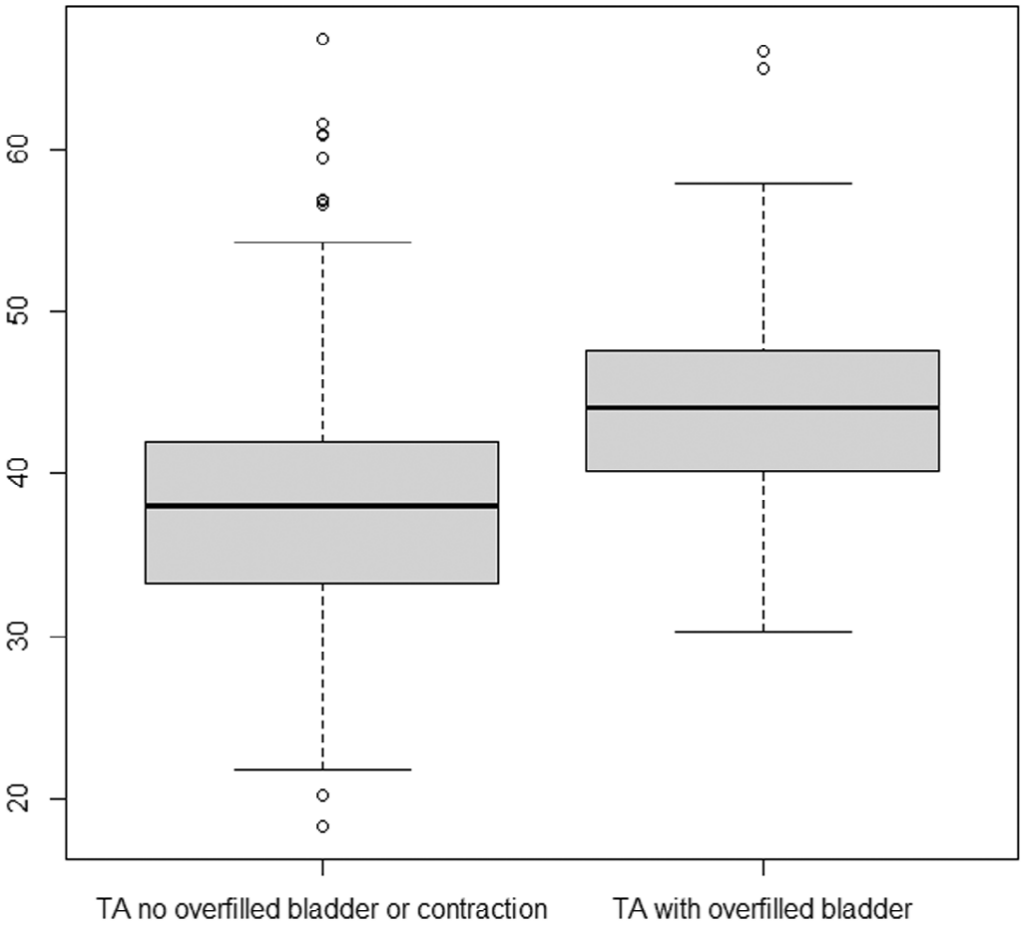
Box‐plot: TA without and overfilled bladder or contraction present compared with TA cervical length with an overfilled bladder (95% CI: 5.2–8 mm). TA, transabdominal.

**Figure 5:**
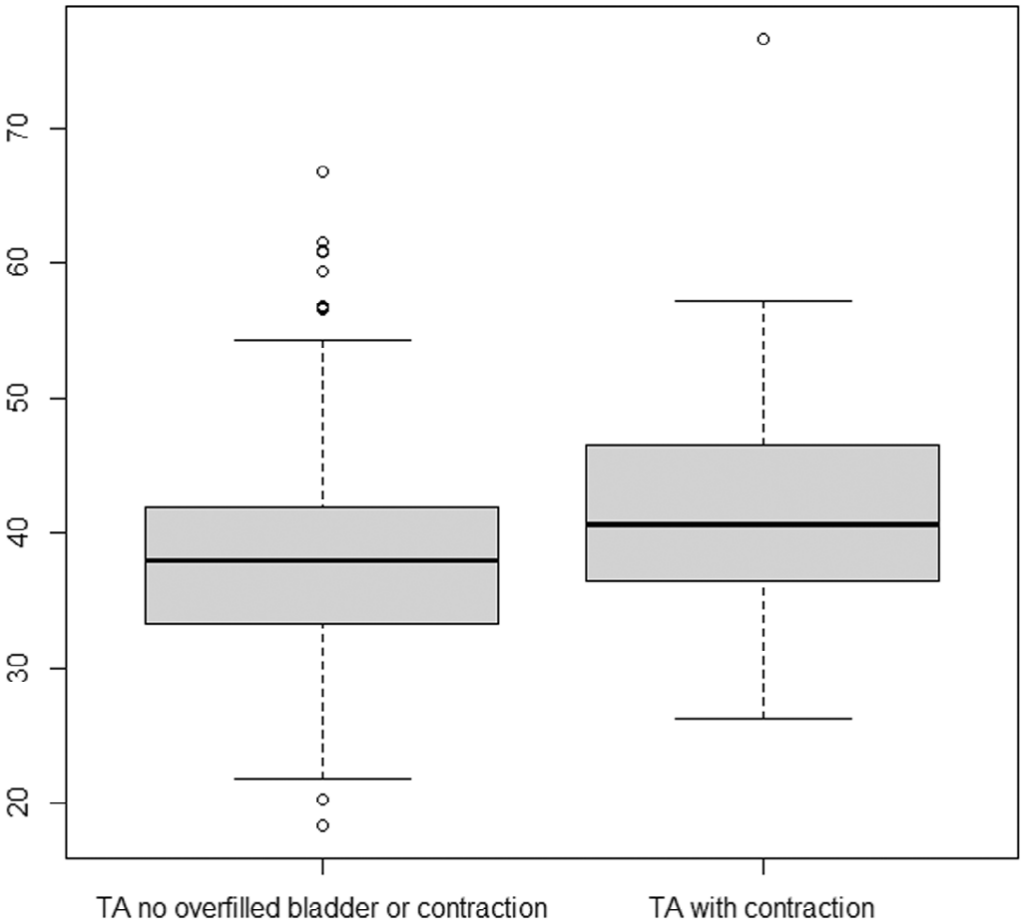
Box‐plot: TA without and overfilled bladder or contraction present compared with TA cervical length with lower uterine contraction (95% CI: 2–4.7 mm). TA, transabdominal.

On TACM, a full bladder and/or lower uterine contraction was seen in 256 patients (30.2%). A total of 118 (13.9%) were classified as having an overfilled bladder, and lower uterine contractions were seen in 156/847 patients (18.4%). The median TACM without overfilled bladder or lower uterine contraction was 38 mm. When an overfilled bladder was present, the median TACM was 44.3 mm (Table 1), with a mean difference of +6.6 mm (95% CI: 5.2–8 mm) (Figure 4). When a lower uterine contraction was present, the median TACM was 40.6 mm, with a mean difference of +3.4 mm (95% CI: 2–4.7 mm) (Figure 5).

**Table 1:** Transabdominal cervical length measurements.

	TA (Total)	No contraction or overfull bladder	Contraction	Overfull bladder	Overfull bladder and contraction
*n*	847	591	138	100	18
Mean (mm)	39.7	38.3	41.6	44.9	44.2
Median (mm)	39.2	38	40.6	44.3	44.3
Range (mm)	18.3–76.7	18.3–66.8	26.2–76.7	30.3–66	32.3–60.4

TA, transabdominal.

**Figure 6:**
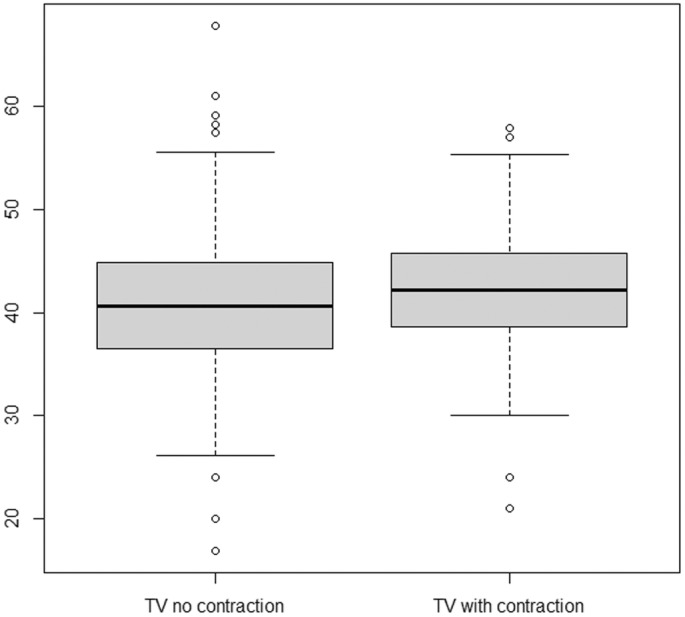
Box‐plot: TV without a contraction present compared with TV cervical length with lower uterine contraction present (95% CI: 0.5–2.4 mm). TV, transvaginal.

On TVCM, bladder fullness was not assessed because the bladder was presumed empty or near empty as patients were directed to empty their bladder directly prior to imaging. Lower uterine contractions were seen in 208 patients of the 711 (29.3%). The median TVCM without a lower uterine contraction was 40.6 mm. The median TVCM with a lower uterine contraction was 42.2 mm (Table 2), with a mean difference of +1.5 mm (95% CI: 0.5–2.4 mm) (Figure 6).

**Table 2:** Transvaginal cervical length measurements.

	TV (Total)	No contraction	Contraction
*n*	711	503	208
Mean (mm)	41.2	40.8	42.3
Median (mm)	41.3	40.6	42.2
Range (mm)	16.9–67.8	16.9–67.8	21–57.9
SD (mm)	6.2	6.2	5.8

TV, transvaginal.

**Figure 7:**
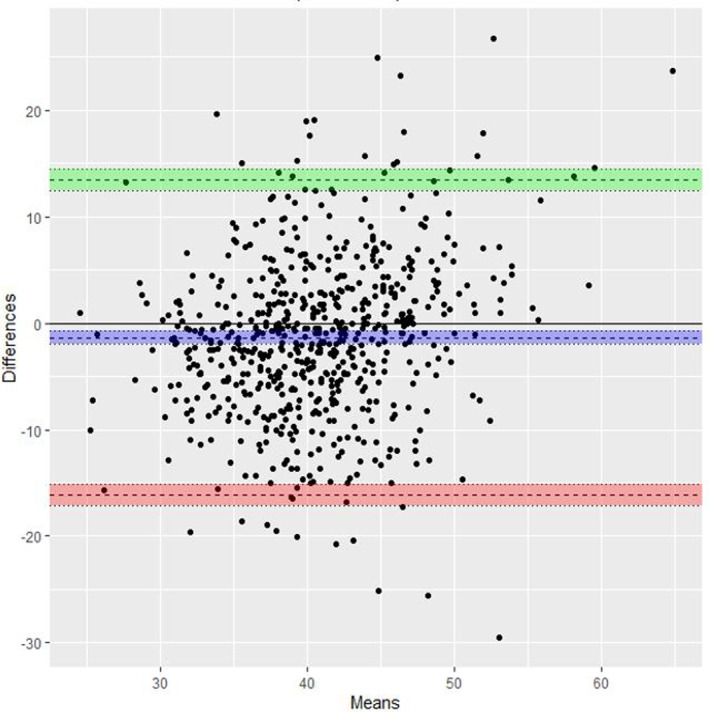
Bland–Altman plot for comparison of TA all and TV all. TA, transabdominal; TV, transvaginal. Green line, Upper limit of agreement with 95% Confidence Interval (CI); Purple line, the difference with 95% CI; Red line, Lower limit of agreement with 95% CI.

**Figure 8:**
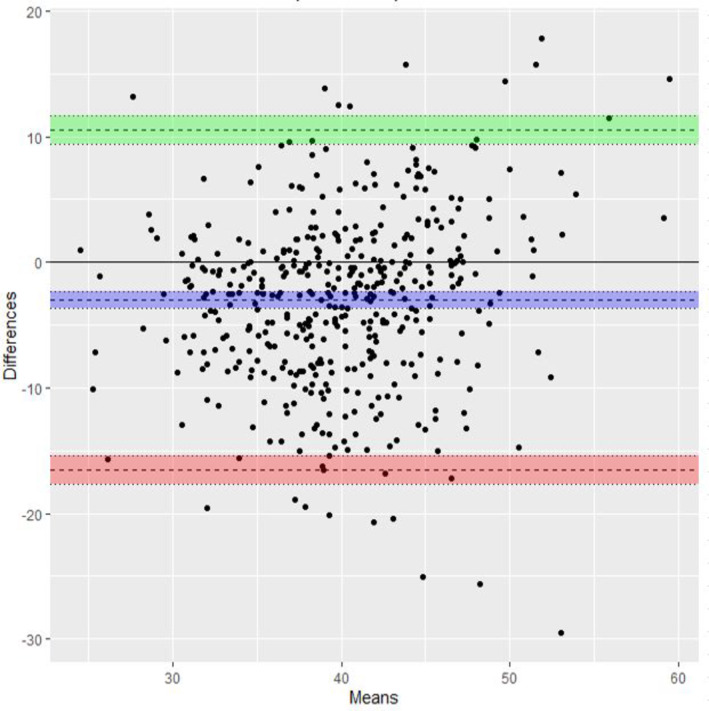
Bland–Altman plot for comparison of TV all and TA no contraction or overfilled bladder. TA, transabdominal; TV, transvaginal. Green line, Upper limit of agreement with 95% Confidence Interval (CI); Purple line, the difference with 95% CI; Red line, Lower limit of agreement with 95% CI.

A comparison of TACM (all) and TVCM (all) can be seen in the Bland–Altman plot (Figure 7). There were 644 TACM and TVCM compared which gave an average discrepancy of −1.35 mm [bias −1.35, limits of agreement (LOA): 13.4, −16.1]. A comparison between TVCM (all) and TACM with no contraction or full bladder present (434 patients) (Figure 8) gave an average discrepancy of −3.02 mm (Bias: −3.02, LOA: 10.5, −16.6).

The single‐score interclass correlation between TACM (all) and TVCM (all) demonstrated poor correlation with an ICC of 0.306 (95% CI: 0.172, 0.429). Single‐score interclass correlation between TVCM (no contractions present) and TACM with no contraction or full bladder present also demonstrated poor correlation with an ICC of 0.379 (95% CI: 0.255, 0.513). There is poor correlation between TACM and corresponding TVCM even with the reduction in variables such as bladder fullness and lower uterine contractions.

**Figure 9:**
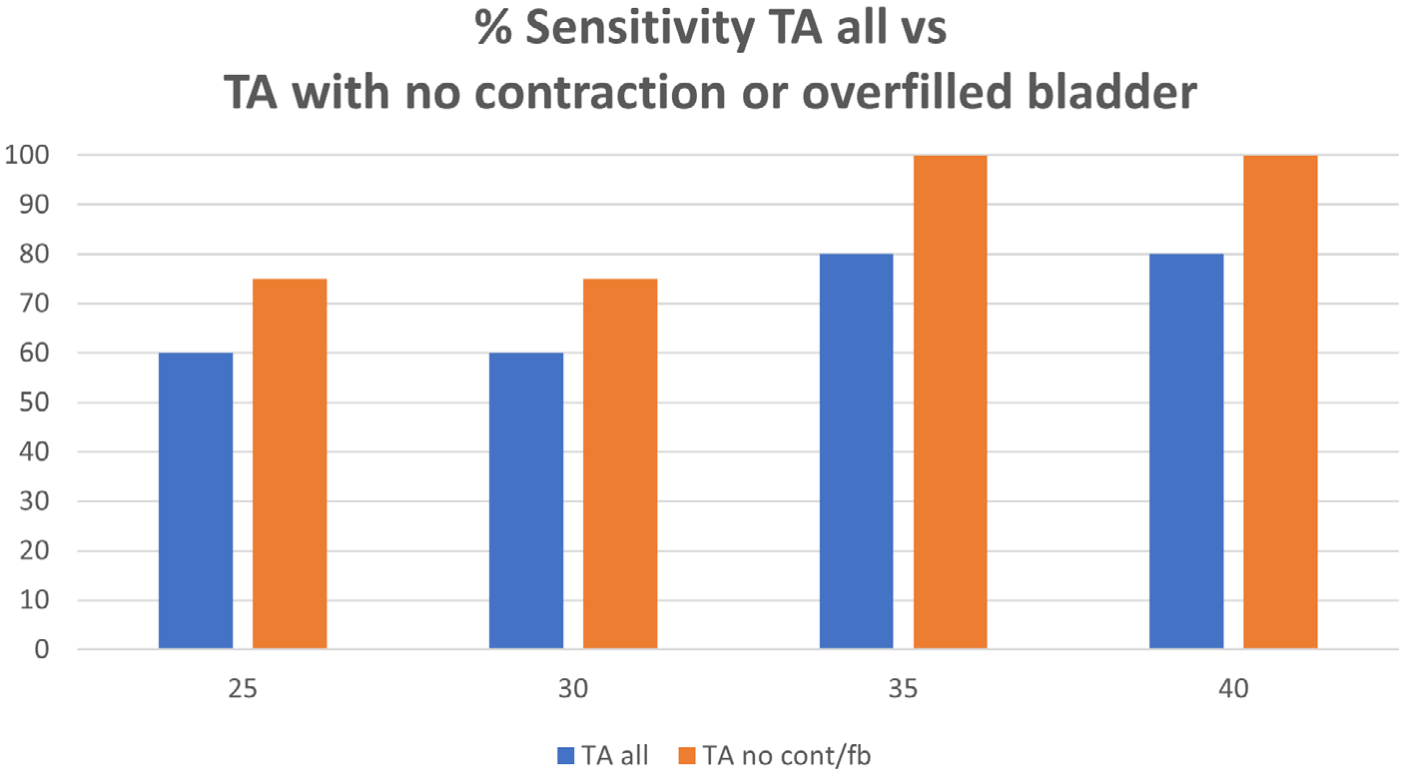
Sensitivity of transabdominal (TA) cervical measurement for detecting a short cervix. TA, transabdominal.

**Figure 10:**
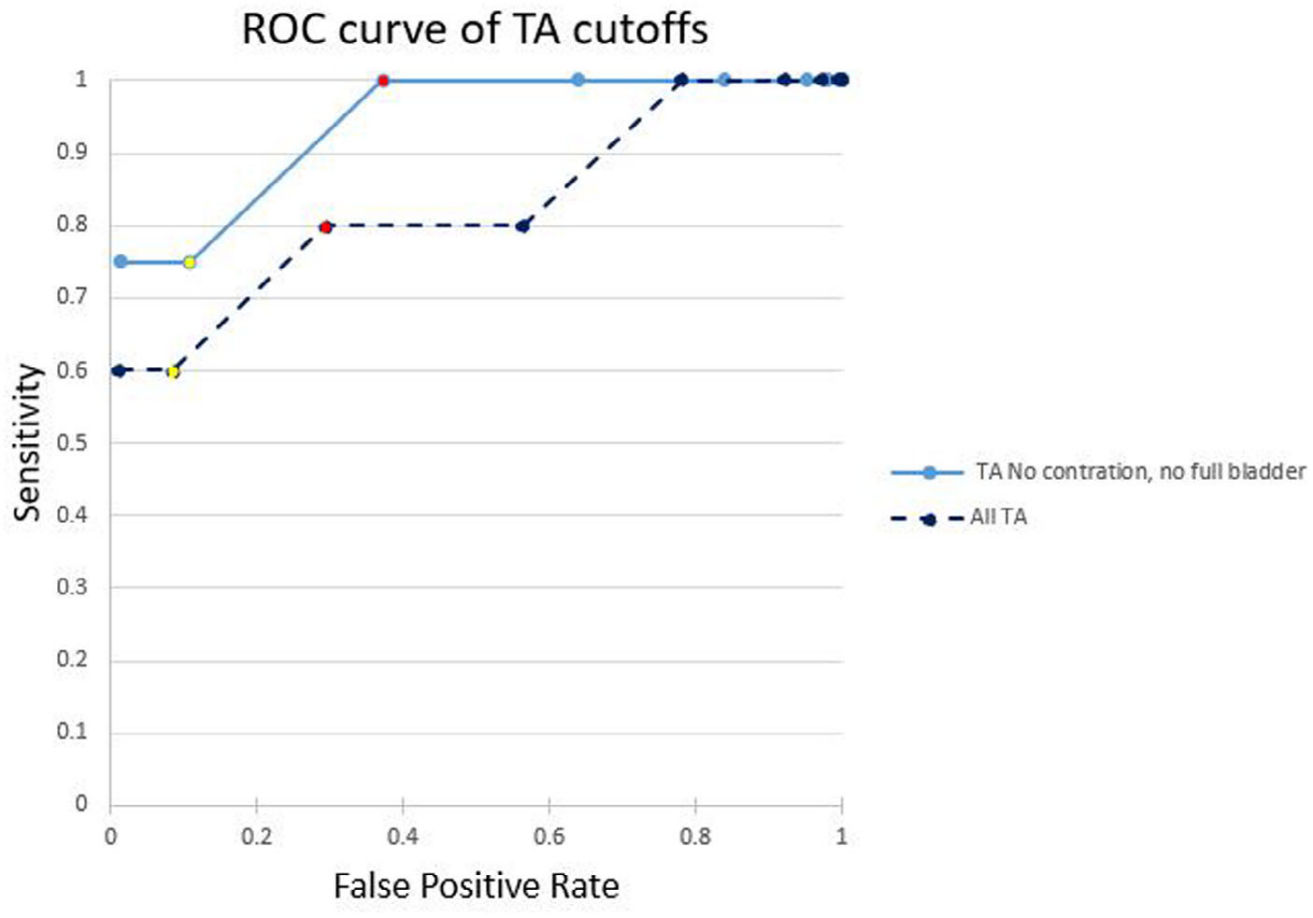
Sensitivity versus false‐positive rate of detecting a short cervix as seen on TV when using a TA cut‐off of ≤25 mm, ≤30 mm (yellow), ≤35 mm (red), ≤40 mm, ≤45 mm, ≤50 mm, ≤55 mm, ≤60 mm and ≤70 mm when measurements are rounded to the nearest 5 mm. TA, transabdominal; TV, transvaginal.

Despite the apparent lack of improved correlation, a higher sensitivity for detecting a short cervix (defined as 25 mm or less on TVCM) was seen at lower cut‐offs when the images did not contain a lower uterine contraction or overfilled bladder (Table 3). Cut‐offs ≤25 mm, ≤30 mm and ≤ 35 mm TACM (all) gave sensitivities of 60%, 60% and 80%, respectively, for detection of a short cervix. When no contractions nor overfilled bladder was present on TACM, the sensitivity at these same cut‐offs was 75%, 75% and 100% (Figure 9). A receiver operator curve demonstrates the potential for an improvement in sensitivity of TACM imaging in detecting a short cervix when no contractions or overfilled bladder is present (Figure 10).

**Table 3:** Sensitivity and specificity of Trans‐abdominal (TA) for detecting a short cervix as defined by 25 mm or less on TV, all and contraction and overfilled bladder excluded (no cont/fb).

TA cut‐off (mm)	Sensitivity	False‐positive rate	Specificity	PPV	NPV
25	60	1	98.9	30	99.7
25 no cont/fb	75	1	98.6	33	99.8
30	60	8	92	5	99.7
30 no cont/fb	75	11	89	6	99.7
35	80	29	71	2	99.8
35 no cont/fb	100	37	62	2	100
40	80	56	44	1	99.6
40 no cont/fb	100	84	36	1	100

NPV, negative predictive value; PPV, positive predictive value.

## Discussion

The impact of an overfilled bladder and lower uterine contractions on cervical length measurements appear to be more apparent on TACM than TVCM. An overfilled bladder and lower uterine contractions appear to lengthen the mean length of the cervix modestly by 6.6 and 3.4 mm, respectively, on TACM. By contrast, lower uterine contractions appear to have minimal impact on TVCM with a mean cervical length increase of only 1.5 mm. When measuring the cervix transvaginally, the presence of a lower uterine contraction is less important and rescanning when the contraction has subsided is usually not necessary. This is because the higher resolution of transvaginal scanning allows recognition of the cervical mucosa as a means of identification of the limits of the cervix even in the presence of a contraction (Figure 2b).

Transabdominal cervical measurements in the absence of an overfilled bladder or contraction improved the bias for the measurement marginally from −1.35 to −3.02, but this is probably not clinically significant. Removing these variables does not improve correlation between TACM and TVCM but appears to improve the sensitivity of TACM to detect a short cervix. When performing a TACM, note should be taken of whether the cephalad margin of the bladder wall is above the level of internal os and whether there is the presence of a lower uterine contraction. If either is present, steps should be taken to remove these confounding factors, for example, emptying the bladder or waiting for resolution of uterine contraction before recording the measurement.

With almost one in five patients in our population declining transvaginal assessment, it would be beneficial to refine the performance of TACM as a screening tool. Correction of confounders such as an overfull bladder and uterine contractions significantly improved the sensitivity of TACM in detecting a short cervix. Our study shows a cut‐off ≤35 mm would provide 100% sensitivity when there is an absence of an overfilled bladder or lower uterine contraction on TACM. Using this cut‐off, 39% of patients would need TVCM when with two‐stage approach is used. In line with our findings, the Royal Australian and New Zealand College of Obstetricians and Gynaecologists suggest TACM can be used for cervical length screening in the mid‐trimester but recommends a TVCM be made when TACM is 35 mm or less or when the cervix cannot be clearly seen transabdominally.^27^

We found 5/453 (1.1%) of women who had a TVCM at 19 weeks gestation had a cervix measuring <25 mm, a prevalence similar to other studies.^11^, ^28^ This low prevalence increases susceptibility of calculations of sensitivities to statistical error. Future studies with larger numbers would help extrapolate reliable calculations.

This study has presented a simple way of assessing bladder fullness which can be easily clinically implemented. The effects of lower uterine contractions on cervical length measurements have not previously been reported in literature. As a retrospective imaging study, there were limited data regarding potential obstetric and demographic risk factors (such as BMI, smoking, and previous obstetric history) and as such were not explored in this study. Transperineal/translabial imaging of the cervix was also not investigated in this study but is an option for patients who decline TVCM or when deemed unsafe (e.g. preterm premature rupture of membranes, open cervix, and recent cerclage). In high‐risk patients such as patients with a history of preterm birth, cervical intraepithelial neoplasia and previous incisional treatment, and women symptomatic of preterm labour, TVCMs are recommended.^27^ However, for low‐risk patients and patients who decline TVCM, TACM can be a feasible method for cervical length screening when confounding factors are removed.

## Conclusion

The novel tool for assessing bladder fullness outlined in this article is useful and easy to implement. TACM is a good screening tool for low‐risk patients and accuracy can be improved when there is use of the novel tool to assess bladder fullness and the presence of lower uterine contractions is taken into consideration. This study has demonstrated that although there is poor correlation between TACM and TVCM, a cut‐off of ≤35 mm when no contractions nor overfilled bladder is present would provide 100% sensitivity with a screen‐positive rate (and progression to transvaginal examination rate) of 39%. TACM ultrasound is useful as part of a two‐stage screening test with the advantage of avoiding TVCM in the majority of patients, hence reducing time taken, cost and patient uneasiness. Future studies using this novel tool with a greater number of proven positives would help validate the data presented in this study.

## Authorship statement

The authors, Heidi Beaver, Gary Low and Valeria Lanzarone, hereby declare that this submission is entirely our own work, in our own words, and that all sources used in researching it are fully acknowledged and all quotations properly identified. The authorship listing conforms to the journal's authorship policy, and all authors are in agreement with the content of the submitted manuscript.

## Funding

No funding was received for conducting this study.

## Conflict of Interest

The authors have no conflicts of interest to declare that are relevant to the content of this article.
